# When dysbiosis meets dystrophy: an unwanted gut‐muscle connection

**DOI:** 10.15252/emmm.202217324

**Published:** 2023-02-27

**Authors:** Anusha Jayaraman, Sven Pettersson

**Affiliations:** ^1^ ASEAN Microbiome Nutrition Centre National Neuroscience Institute Singapore Singapore; ^2^ Faculty of Medical Sciences Sunway University Subang Jaya Malaysia; ^3^ Department of Microbiology and Immunology National University of Singapore Singapore Singapore; ^4^ Department of Odontology Karolinska Institutet Stockholm Sweden

**Keywords:** Digestive System, Musculoskeletal System

## Abstract

Duchenne muscular dystrophy (DMD) is a devastating neuromuscular degenerative disease with no known cure to date. In recent years, the hypothesis of a “gut‐muscle axis” has emerged suggesting that bidirectional communication between the gut microbiota and the muscular system regulates the muscular function and may be perturbed in several muscular disorders. In addition, the excessive consumption of sugar and of lipid‐rich processed food products are factors that further aggravate the phenotype for such diseases and accelerate biological aging. However, these unhealthy microbiota profiles can be reversed by individualized dietary changes to not only alter the microbiota composition but also to reset the production of microbial metabolites known to trigger beneficial effects typically associated with prolonged health span. Two recent studies (in this issue of EMBO Mol Med) highlight the interesting potential of microbiota‐informed next‐generation dietary intervention programs to be considered in genetically linked muscle disorders like DMD.

## Graphical abstract

A. Jayaraman and S. Pettersson discuss the studies by Farini *et al* and Kalkan *et al*, in this issue of EMBO Mol. Med., that independently reported the potential role of the gut microbiota in the pathogenesis of Duchenne muscular dystrophy.

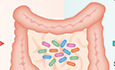

Duchenne muscular dystrophy (DMD) is an X‐linked genetic mutation disorder, characterized by progressive muscle wasting and dysfunctional intestinal functions leading to severe disability and shortened lifespan. DMD affects multiple organ systems including heart and lung muscles further aggravating the physiology of the patient. DMD is caused by a mutation in the dystrophin gene, coding for a protein in a protein complex that strengthens muscle fibers and protects them from injury during muscle contraction and relaxation. Sadly, treatment options for DMD are limited to glucocorticoids to slow the progression of the disease. Management strategies include physiotherapeutic interventions focussing on breathing and swallowing, and dietary guidance to improve swallowing. Other emerging treatment strategies under evaluation include gene therapy, antisense oligonucleotides to induce exon skipping, stem cell and other cell‐based therapies, anti‐inflammatory drugs, vasodilation, and antifibrotic treatment, among others (Markati *et al*, 2022).

The gut microbiota composition and microbial metabolites are being investigated in several disease contexts due to the bidirectional communication between gut microbes and their host. Gut microbes process either dietary components or host‐derived substrates to produce metabolites (e.g., short‐chain fatty acids [SCFA] and bile acids). This process is highly dynamic and linked to dietary fluctuations and microbiome composition (Nicholson *et al*, 2012). Gut microbes have coevolved with their hosts to regulate vital life functions, influencing several distal organs through their metabolites via the circulatory and lymphatic systems. Thus, studies have shown that perturbations to this complex relationship through dysbiosis are likely a critical underlying factor in several diseases, including muscular disorders, in an age‐ and sex‐dependent manner (Bellini *et al*, 2006; Lahiri *et al*, 2019; DeJong *et al*, 2020).

In the last 10 years, several publications have firmly established that when an organ pathologically declines in the host, long‐lasting alterations of gut microbiota composition occur, often resulting in dysbiosis, worsening in turn the disease symptoms (DeJong *et al*, 2020). Along these lines, two recent studies (Farini *et al*, 2022; Kalkan *et al*, 2023) report on the role of gut dysbiosis in a DMD mouse model, the mdx dystrophic murine model (Fig 1).

**Figure 1:**
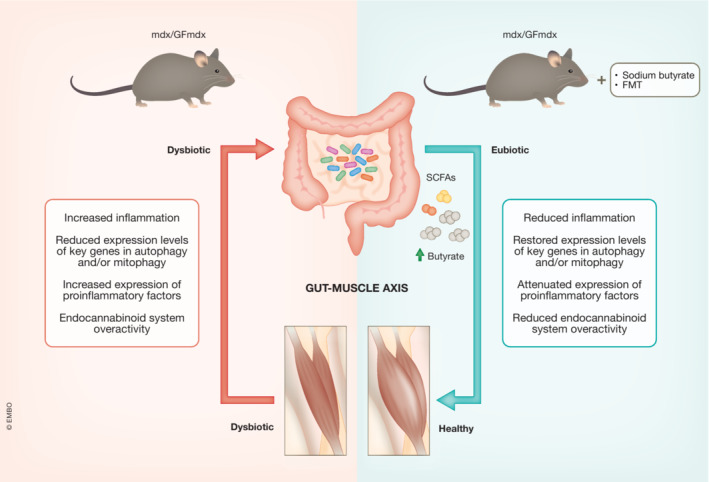
The gut‐muscle axis The diagram illustrates the interaction between gut microbiota and the muscles to either aggravate or ameliorate inflammation and organ health. FMT, fecal microbiota transplant; GF, germ‐free; SCFAs, short‐chain fatty acids.

Kalkan *et al* show that the gut microbiota composition differed significantly between wildtype and mdx mice. In addition, when mdx mice were treated with deflazacort (DFZ), a synthetic glucocorticoid used for DMD treatment, their gut microbiota composition clustered with that of the wildtype mice, suggesting that the use of a metabolically supporting anti‐inflammatory drug could revert the microbiome composition in the mutant mice. Interestingly, they highlighted an enrichment of the *Prevotellaceae* family in the mdx mice, also observed independently by Farini *et al* (2022).

At the molecular level, previous data had suggested potential beneficial effects of using SCFA to improve muscle function in germ‐free mice (Lahiri *et al*, 2019). Interestingly, among the microbial metabolites that differed between wildtype and mdx groups, SCFA and key precursors in the biosynthesis pathway of SCFA were found to be significantly reduced in the plasma. Notably, in addition to reversing deficits in locomotor activity, SCFA levels, muscle autophagy, and inflammation, Kalkan *et al* show that DFZ treatment induced a remarkable increase in the concentration of SCFA, butyrate, known to have beneficial roles including anti‐inflammatory and immunomodulatory properties (Fernandes *et al*, 2020; Kalkan *et al*, 2023).

The Iannotti group had previously shown that 19‐week‐old mdx mice displayed significant impairment in muscle strength and coordination (Iannotti *et al*, 2018). Assuming that decrease in butyrate levels observed in the mdx mice might contribute to the muscle phenotype, the Kalkan team orally supplemented the mice with sodium butyrate. Remarkably, butyrate supplementation was able to reduce locomotor deficits in these mice. Additionally, butyrate also restored the expression levels of key genes in autophagy and/or mitophagy, attenuated the expression of several proinflammatory factors, and reduced endocannabinoid system overactivity in mdx mice. The authors corroborated their findings by ex vivo experiments using lipopolysaccharide (LPS) to reproduce the inflammation microenvironment in murine C2C12 skeletal muscle cells. Butyrate treatment reduced LPS‐induced inflammation and endocannabinoid overactivity, and stimulated autophagy through G‐protein couple receptor (GPR109A) and PPARγ. Strikingly similar results were obtained in primary muscle cells from DMD donors.

In a broader context, the results presented in this paper demonstrate the potential of using SCFA as an intervention strategy for DMD and may apply to other age‐related diseases such as type 2 diabetes, hypertension, cancer, cardiovascular diseases, and neurodegenerative disorders, which have also been reported to respond positively to SCFA intervention therapy (Fernandes *et al*, 2020).

The second study, by Farini *et al*, uses a different approach to assess the gut microbiota dysbiosis in DMD, particularly focussing on the immune response associated with muscle inflammation. The study showed that the enrichment of *Prevotella* in mdx mice was correlated with different subsets of T cells in the spleen and muscle. By using broad‐spectrum antibiotics treatment in mdx mice, gut microbes were depleted, accompanied by a reduction in chronic muscle inflammation, thus additionally linking the dysbiotic microbiome composition and the phenotypic symptoms observed in mdx mice.

In addition, they also performed a fecal transplant experiment in which mdx mice received eubiotic microbiota. They observed a reduction in the muscle inflammatory response and a reversal of other deficits in muscle pathology and function such as decreased myofiber cross‐sectional area, fibrotic infiltrate, decreased lipid and carbohydrate metabolism, and reduced tetanic force of tibialis anterior muscle in mdx mice.

Given that gastrointestinal and metabolic issues are common in patients with DMD, these two studies unfold the possibility of using gut microbiota metabolite intervention strategies for managing or reversing DMD symptoms (Farini *et al*, 2022; Kalkan *et al*, 2023). If the use of SCFA or molecules stimulating the production of SCFA can be shown to have beneficial effects in patients with DMD, they may increase the arsenal of therapies that may slow down the progression of DMD and complement steroid treatment. Both studies demonstrate a significant role for gut microbiota in DMD, particularly with changes in the microbiota composition and microbial metabolites such as SCFA. Because diet is a major factor in modifying gut microbiota diversity and gut microbes are malleable and respond to dietary interventions, these studies also open the possibility for future food intervention therapies to stimulate an increase in gut microbes capable of producing SCFA.

While these data are limited to an animal model of DMD, they highlight the importance of generating detailed microbiota information, pertaining not only to the microbiota composition itself but also to the levels of SCFA among the individual patients with DMD. In essence, a tailor‐made intervention strategy could be envisaged, well in line with the vision and mission of 21^st^ century precision medicine.

## Author contributions

**Anusha Jayaraman:** Conceptualization; writing – original draft; writing – review and editing. **Sven Pettersson:** Writing – review and editing.

## References

[emmm202217324-bib-0001] Bellini M , Biagi S , Stasi C , Costa F , Mumolo MG , Ricchiuti A , Marchi S (2006) Gastrointestinal manifestations in myotonic muscular dystrophy. World J Gastroenterol 12: 1821–1828 16609987 10.3748/wjg.v12.i12.1821 PMC4087506

[emmm202217324-bib-0002] DeJong EN , Surette MG , Bowdish DME (2020) The gut microbiota and unhealthy aging: disentangling cause from consequence. Cell Host Microbe 28: 180–189 32791111 10.1016/j.chom.2020.07.013

[emmm202217324-bib-0003] Farini A , Tripodi L , Villa C , Strati F , Facoetti A , Baselli G , Troisi J , Landolfi A , Lonati C , Molinaro D *et al* (2022) Microbiota dysbiosis influences immune system and muscle pathophysiology of dystrophin‐deficient mice. EMBO Mol Med 15: e16244 36533294 10.15252/emmm.202216244 PMC9994487

[emmm202217324-bib-0004] Fernandes MF , de Oliveira S , Portovedo M , Rodrigues PB , Vinolo MAR (2020) Effect of short chain fatty acids on age‐related disorders. Adv Exp Med Biol 1260: 85–105 32304031 10.1007/978-3-030-42667-5_4

[emmm202217324-bib-0005] Iannotti FA , Pagano E , Guardiola O , Adinolfi S , Saccone V , Consalvi S , Piscitelli F , Gazzerro E , Busetto G , Carrella D *et al* (2018) Genetic and pharmacological regulation of the 824 endocannabinoid CB1 receptor in Duchenne muscular dystrophy. Nat Commun 9: 3950 30262909 10.1038/s41467-018-06267-1 PMC6160489

[emmm202217324-bib-0006] Kalkan H , Pagano E , Paris D , Panza E , Cuozzo M , Moriello C , Piscitelli F , Abolghasemi A , Gazzerro E , Silvestri C *et al* (2023) Targeting gut dysbiosis against inflammation and impaired autophagy in Duchenne muscular dystrophy. EMBO Mol Med 15: e16225 36594243 10.15252/emmm.202216225 PMC9994484

[emmm202217324-bib-0007] Lahiri S , Kim H , Garcia‐Perez I , Reza MM , Martin KA , Kundu P , Cox LM , Selkrig J , Posma JM , Zhang H *et al* (2019) The gut microbiota influences skeletal muscle mass and function in mice. Sci Transl Med 11: eaan5662 31341063 10.1126/scitranslmed.aan5662 PMC7501733

[emmm202217324-bib-0008] Markati T , Oskoui M , Farrar MA , Duong T , Goemans N , Servais L (2022) Emerging therapies for Duchenne muscular dystrophy. Lancet Neurol 21: 814–829 35850122 10.1016/S1474-4422(22)00125-9

[emmm202217324-bib-0009] Nicholson JK , Holmes E , Kinross J , Burcelin R , Gibson G , Jia W , Pettersson S (2012) Host‐gut microbiota metabolic interactions. Science 336: 1262–1267 22674330 10.1126/science.1223813

